# Spleen Tyrosine Kinase Is Involved in the CD38 Signal Transduction Pathway in Chronic Lymphocytic Leukemia

**DOI:** 10.1371/journal.pone.0169159

**Published:** 2016-12-30

**Authors:** Marco Benkisser-Petersen, Maike Buchner, Arlette Dörffel, Marcus Dühren-von-Minden, Rainer Claus, Kathrin Kläsener, Kerstin Leberecht, Meike Burger, Christine Dierks, Hassan Jumaa, Fabio Malavasi, Michael Reth, Hendrik Veelken, Justus Duyster, Katja Zirlik

**Affiliations:** 1 Department of Hematology, Oncology and Stem cell transplantation, University Medical Center, Faculty of Medicine, University of Freiburg, Freiburg, Germany; 2 Faculty of Biology, University of Freiburg, Freiburg, Germany; 3 Institute for Clinical Chemistry and Pathobiochemistry, Technische Universität München, München, Germany; 4 Department of Internal Medicine V, Heidelberg University Hospital, Heidelberg, Germany; 5 Department of Immunology, University Medical Center, Ulm, Germany; 6 BIOSS Centre for Biological Signalling Studies, Department of Molecular Immunology, Biology III, University of Freiburg, Freiburg, Germany; 7 Max Planck Institute of Immunobiology and Epigenetics, Freiburg, Germany; 8 Department of Medical Sciences, Laboratory of Immunogenetics and CeRMS, University of Torino, and Transplant Immunology, Torino, Italy; 9 Department of Hematology, Leiden University Medical Center, Leiden, The Netherlands; University of Kentucky, UNITED STATES

## Abstract

The survival and proliferation of CLL cells depends on microenvironmental contacts in lymphoid organs. CD38 is a cell surface receptor that plays an important role in survival and proliferation signaling in CLL. In this study we demonstrate SYK's direct involvement in the CD38 signaling pathway in primary CLL samples. CD38 stimulation of CLL cells revealed SYK activation. SYK downstream target AKT was subsequently induced and MCL-1 expression was increased. Concomitant inhibition of SYK by the SYK inhibitor R406 resulted in reduced activation of AKT and prevented upregulation of MCL-1. Moreover, short-term CD38 stimulation enhanced BCR-signaling, as indicated by increased ERK phosphorylation. CXCL12-dependent migration was increased after CD38 stimulation. Treating CLL cells with R406 inhibited CD38-mediated migration. In addition, we observed marked downregulation of CD38 expression for CLL cells treated with R406 compared to vehicle control. Finally, we observed a clear correlation between CD38 expression on CLL cells and SYK-inhibitor efficacy. In conclusion, our study provides deeper mechanistic insight into the effect of SYK inhibition in CLL.

## Introduction

B-cell chronic lymphocytic leukemia (CLL) is one of the most prevalent B-cell malignancies in adults and is characterized by the expansion of monoclonal mature B-cells. The highly variable prognosis of this disease may be predicted using a number of biomarkers, including CD38 expression level.[1] Human CD38 is a transmembrane glycoprotein that catalyzes the synthesis of cyclic ADP ribose (cADPR), an important second messenger mobilizing Ca^2+^ from Ryanodine-sensitive intracellular stores.[2,3] CD38 has also the ability to mediate cell-cell interactions by binding the non-substrate ligand CD31 (PECAM-1, a member of the Ig superfamily), which is expressed on endothelial cells, nurse-like cells, and CLL cells. CD38 is expressed on a variety of cell types including immature B-lymphocytes and plasma cells. CD38 expression varies in CLL and there is evidence that CD38 expression is induced in so-called pseudofollicles, the proliferative compartment of CLL.[4] Analysis of CD38 gene polymorphisms revealed a functional link with CLL disease progression and the risk of Richter transformation.[5] Furthermore, high CD38 expression is associated with a poor response to chemotherapy and reduced survival.[1] In recent years, CD38 has also been recognized as a potential therapeutic target. Several CD38 monoclonal antibodies for use in hematological malignancies are currently under investigation in clinical trials.

*In vitro* studies have shown that the signaling induced downstream of the CD38 molecule has a pro-survival and proliferative function.[6] Furthermore, CD38/CD31 interactions increase CXCL-12-mediated signals and the homing of CLL cells towards lymphoid organs.[7] CD38 also associates with the CD49d/CD29 complex and enhances integrin-mediated F-actin polymerization, cell adhesion, and apoptosis resistance.[8] Additionally, the involvement of CD38 in B-cell receptor (BCR) signaling has been proposed as the molecule associates with the BCR complex in lipid rafts and causes the activation of BCR components.[9–11]

The interaction of CD38 with BCR signaling may also be of clinical interest, as novel treatment strategies focusing on the inhibition of BCR pathway components like BTK and PI3 kinase δ have proven efficacy in CLL.[12,13]

The ligation of CD38 induces tyrosine phosphorylation of several intracellular proteins, including spleen tyrosine kinase (SYK) in immature B-cells as well as in lamina propria T-cells.[14,15] We and others previously identified SYK as a candidate for targeted therapy in CLL due to its enhanced expression and activity and the apoptotic effects of pharmacological SYK inhibition.[16,17] Of note, ongoing clinical trials are continuing to investigate the selective SYK inhibitor entospletinib. Very recently, entospletinib demonstrated promising clinical activity in patients with relapsed or refractory CLL.[18] Given the association between SYK and CD38 in lymphoid cells, we aimed to determine whether SYK is involved in CD38 signaling in CLL cells and may represent a potential target to prevent CD38-mediated CLL cell survival, and migration.

## Materials and Methods

### Patients and cell lines

This study was approved by the Institutional Review Board of University Medical Center Freiburg. After written informed consent, peripheral blood samples were obtained from patients at Freiburg University Hospital fulfilling diagnostic and immunophenotypic criteria for CLL. All patients were either untreated or had been off therapy for at least 6 months before the study started. The patient characteristics including age at diagnosis, Binet stage, Rai stage, IgVH (immunoglobulin heavy-chain variable-region) mutation status, genomic aberrations, and ZAP70 status are summarized in Table 1. Peripheral blood mononuclear cells (PBMCs) were separated by Ficoll gradient centrifugation. From CLL samples containing less than 80% B CLL cells as determined by flow cytometric analysis, CLL cells were isolated by negative selection using the CLL Isolation Kit (Miltenyi Biotech, Auburn, CA), according to the manufacturer's instructions. Cells were either used fresh or resuspended in fetal calf serum (FCS) with 5% dimethyl sulfoxide (DMSO) and stored in liquid nitrogen until use.

**Table 1:** Patient characteristics (including Age at diagnosis, Binet stage, Rai stage, IgVH mutation status (um indicates unmutated, m mutated), genomic aberrations such as deletion 11q, deletion 13q14, deletion 17p, trisomy 12, ZAP70 status (pos indicates ZAP70 expression > 20%, neg indicates ZAP70 expression < 20%) (n.d. indicates not done, 1 indicates variable present, 0 indicates variable not present).

ID	Sex	Age at diagnosis	Binet	Rai	IgVH mutation	Del 11q	Del 13q14	Del 17p	Trisomy12	ZAP70
1	m	73	C	III	um	1	1	0	0	pos
2	m	66	A	0	um	0	1	0	0	neg
3	m	44	B	II	um	0	1	0	0	pos
4	m	66	B	II	m	0	1	0	1	neg
5	m	66	A	I	um	1	1	0	0	pos
6	m	58	A	I	m	0	0	0	0	neg
7	m	52	C	IV	um	0	1	1	0	pos
8	m	40	B	I	um	0	0	0	0	pos
9	m	74	n.d	n.d	m	0	0	0	0	neg
10	m	58	n.d	n.d	um	0	1	1	0	pos
11	m	63	n.d	n.d	n.d	0	0	0	0	n.d.
12	f	62	B	III	n.d	0	1	0	0	n.d.
13	m	49	B	II	um	1	1	0	0	pos
14	m	47	B	II	m	0	1	0	1	neg
15	m	76	C	IV	n.d	0	1	0	0	n.d.
16	m	78	A	0	n.d	0	0	0	1	n.d.
17	m	67	C	III	m	0	1	0	0	neg
18	m	42	B	II	um	1	1	0	0	pos
19	m	60	B	III	um	0	1	0	0	n.d.
20	m	50	C	IV	m	1	0	0	0	neg
21	m	71	B	II	n.d	n.d	n.d	n.d	n.d	n.d.
22	f	65	B	II	m	0	1	0	1	neg
23	m	57	B	II	m	0	1	0	0	neg
24	m	80	C	III	um	0	0	0	1	n.d.
25	m	67	n.d	nd	um	1	1	0	0	n.d.
26	f	60	A	I	um	0	0	0	0	pos
27	f	68	A	I	n.d	n.d	n.d	n.d	n.d	n.d.
28	f	53	C	IV	um	0	1	0	0	pos
29	m	50	C	IV	m	1	0	0	0	neg
30	m	51	B	II	m	0	1	0	1	neg

### CLL cell stimulation

For CD38 stimulation, primary CLL cells were incubated at 5*10^6^ cells/ml in 24-well plates, which were pre-coated with 3.3 μg/ml CD31/PECAM (R&D Systems, Minneapolis, MN) over night at 4°C and then washed twice with PBS. Alternatively, CLL cells were stimulated with IB4, an agonistic anti-CD38 mAb produced by one of the authors, at a concentration of 5–8 μg/ml for the indicated time frames. IgG2a (BD Pharmingen) was used as an appropriate isotype control.

For CD40 stimulation, CLL cells were incubated with 1 μg/ml CD40 ligand (CD40L) for 24 hs (R&D Systems). For CXCR4 stimulation, primary CLL cells were incubated with 200 ng/ml CXCL12 (R&D Systems) for the indicated time frames. For pharmacological SYK inhibition, primary CLL cells were treated with 4 μM R406 or 2 μM P505-15 (both Selleckchem, Houston, TX) 30 min prior to stimulation experiments.

### Analysis of the SYK phosphorylation site Y^352^

The phosphorylation status of SYK Y^352^ was measured by flow cytometry. CLL cells were centrifuged at 100 g for 10 sec onto wells pre-coated with CD31/PECAM and incubated for 15 sec for phospho-SYK (pSYK) detection. Then, cells were fixed, washed, permeabilized (using components of the Foxp3 staining kit (ebioscience, San Diego, CA), and incubated with a primary pSYK^Y352^ antibody (Cell Signaling Technology, Danvers, MA). Cells were then stained with a FITC-labelled anti-rabbit F(ab')_2_ IgG fragment (Jackson Immunoresearch, West Grove, PA) and analyzed using a Cyan ADP cytometer (Beckman Coulter, Fullerton, CA). Data were analyzed using FlowJo software (Tree Star, San Carlos, CA).

### Immunoblotting

Total cell protein was extracted as described [19] and total protein concentration was determined using BCA Protein Assay kit (Pierce, Rockford, IL). Proteins were separated by SDS-polyacrylamide gel electrophoresis (SDS-PAGE) and transferred to a polyvinylidene difluoride (PVDF) membrane. Membranes were incubated at 4°C over night with primary antibodies to SYK, pSYK^Y525/526^, pAKT^S473^, pERK 1/2^T202/Y204^, MCL1, β-actin (all Cell Signaling Technology) or GAPDH (Santa Cruz Biotechnology, Dallas, TX). Binding of secondary horseradish peroxidase-conjugated antibodies (Cell Signaling Technology; Sigma Aldrich, St. Louis, MO) was detected using enhanced chemiluminescence and autoradiography with hyperfilm ECL (Amersham Biosciences, Buckinghamshire, UK). Densitometric analysis of immunoblots was performed using ImageJ software.

### Quantitative PCR

Quantitative PCR was performed by standard procedures. Briefly, total RNA from 5*10^6^ primary CLL cells was isolated using the RNeasy Blood and tissue kit (Qiagen, Hilden, Germany). Subsequently, 500 ng of RNA were transcribed to cDNA using oligo dT primers (Superscript III, Thermo Fisher Scientific). Q-PCR was performed using specific primers for CD38, E2A and NF-kB. ERCC6 was used as loading control.

Primers used were: CD38 forward: 5´-CTGAGGATTCATCTTGCACATCTG-3´, CD38 reverse: 5´-GGCTTCCGTCTCTGGCATTG-3´, NFKB1 forward: 5´-GAAGCTGAAGTGCATCCAAAGG-3, NFKB1 reverse: 5´-GCCAGTGTTGTGATTGCTAGATAC-3´, E2A forward: 5´-TCCCTTCTCGGTGGCTTCC-3´, E2A reverse: 5´-CGCACAGTTCCAGAGGCTATG-3´. A reference gene assay was used for ERCC6 (Primerdesign Ltd., Southampton, UK).

### Analysis of CLL cell migration

CLL cells were treated with 4 μM R406 or DMSO for 30 min at 37°C and subsequently incubated with IB4 mAb (8 μg/ml) or isotype control for 30 min at 4°C. Cells were then transferred to the upper compartment of a transwell chamber containing CXCL12 (200 ng/ml). Cells were further cultured for 2 h and subsequently stained with anti-CD5-APC mAb (BD Biosciences, Franklin Lakes, NJ) and anti-CD19-PE-TxRed mAb (Southern Biotech, Birmingham, AL). The number of migrated CLL cells was measured by flow cytometry. Percentage of migrated CLL cells was calculated as number of migrated CLL cells / total number of CLL cells*100.

### Analysis of apoptosis

CLL cells were cultured in RPMI 1640 medium (Sigma-Aldrich) supplemented with 10% FCS, 100 U/mL penicillin, 0.1 mg/mL streptomycin at a concentration of 5 × 10^6^ cells/mL for 24 h in the presence or absence of 4 μM R406. For apoptosis analysis, CLL cells were stained with Annexin V-FITC (BD Biosciences) and propidium iodide (Sigma-Aldrich). Apoptosis induction was analyzed by flow cytometry.

### CD38 expression

5 x 10^5^ cells were stained with anti-CD5-APC, anti-CD19-PE-Texas Red and anti CD38-FITC (Beckman Coulter) for 30 min at 4°C and washed with PBS. Cells were subsequently analyzed using a Cyan ADP flow cytometer. CD38 surface expression on CLL cells was analyzed by gating on CD5/CD19 double positive cell populations.

### Calcium flux

For Ca^2+^ flux analysis, 1 x 10^6^ CLL cells were loaded with FuraRed (Thermo Fisher Scientific, Waltham, MA). CLL cells were treated with R406 for 30 min and subsequently incubated with IB4 mAb (5 μg/ml for 45 min at 37°C). Ca^2+^ flux was analyzed by flow cytometry as reported before.[20] CD38-dependent induction of Ca^2+^ flux was monitored after incubating cells with IB4. BCR stimulation was induced by addition of goat anti human kappa ab (Southern Biotech).

### Cell sort

Primary CLL cells were incubated with APC-labeled anti-CD38 mAb (BD Biosciences) for 30 min at 4°C. After two rounds of washing with PBS, CD38-positive and negative cells were sorted using an Aria Fusion cell sorter (BD Biosciences).

### Statistical analysis

Statistical analyses were performed using the GraphPad Prism software, version 5.0.

P values <0.05 were considered * significant, values < 0.01 ** significant, and values < 0.001 *** significant.

## Results

### SYK is phosphorylated upon CD31/PECAM contact

**Fig 1:**
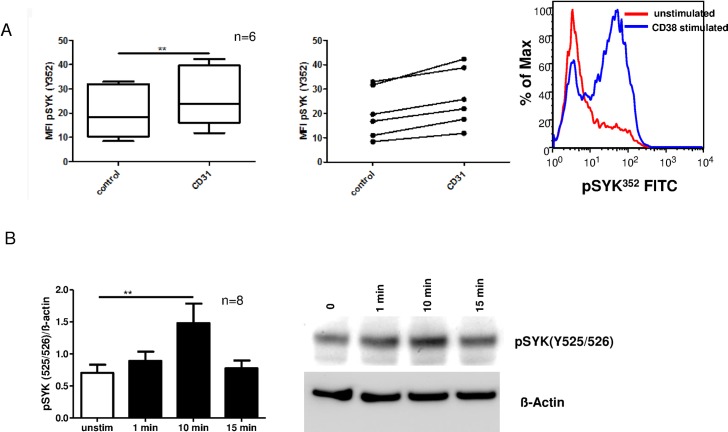
SYK is rapidly induced upon CD38 stimulation. CD38 stimulation of primary CLL cells was performed using recombinant human CD31. A) Initial activation of SYK by phosphorylation of tyrosine residue 352 was detected after exposure of CLL cells to CD31 for 15 sec (n = 6, p<0.01) (left). Middle: Before and After Plot of 6 CLL patients. Right: A representative example of n = 6 independent experiments is shown. B) Trans-autophosphorylation of SYK upon CD38 activation was analyzed by western blot using a SYK Y(525/526) specific antibody. Left: densitometric analysis of pSYK western blots after CD31 ligation for 1 minute, 10, or 15 minutes (n = 8, p<0.01). Error bars indicate SEM. Right: A representative example of n = 8 independent experiments is shown.

First, we analyzed the SYK tyrosine phosphorylation status after CD38 stimulation that has been reported to occur in immature B-cell lines.[15] CD38 ligation was followed by a consistent phosphorylation of the tyrosine residue Y352, the first activation site that releases SYK from its autoinhibitory conformation [21] as determined by flow cytometry (Fig 1A, n = 6, p<0.01). In a larger series of experiments (n = 18) we could not identify prognostic subgroups of patients that showed a more pronounced response than others. Immunoblotting for the SYK autophosphorylation site 525/526 revealed a substantial increase in phosphorylation after 10 min of CD31/PECAM contact (Fig 1B, n = 8, p<0.01). In addition, we investigated SYK phosphorylation after CD31 ligation in the presence or absence of R406 to evaluate true baseline phosphorylation of Y525/526 (S1 Fig).

### SYK mediates CD31/CD38-induced AKT activation and MCL-1 expression in CLL

A functional characterization of the role of SYK in the CD38-CD31 interaction in CLL cells was obtained by monitoring activation of the PI3K pathway in the presence or absence of the SYK inhibitor R406 with and without CD38 stimulation in primary CLL cells.

**Fig 2:**
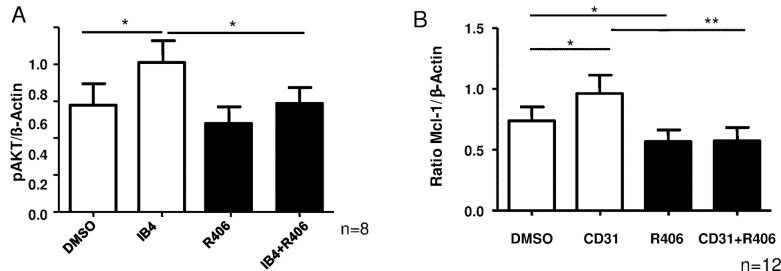
SYK mediates CD38 induced AKT activation and MCL-1 expression. A) Western blot analysis of pAKT (Ser473) induction after incubation of CLL cells with IB4 for 15 min with or without concomitant R406 treatment (n = 8, p<0.05). B) Western Blot analysis of MCL-1 expression after 24 hs of CD31 ligation with or without concomitant SYK inhibition (n = 12, p<0.05 and p<0.01). Error bars indicate SEM.

Previously we could validate SYK inhibitor specificity by performing a protein tyrosine kinase activity assay, demonstrating that the SYK inhibitor R406 only decreased the kinase activity of SYK but not of ZAP70.[16] Phosphoinositide 3-kinase (PI3K) is a downstream target in the CD38 pathway and its activity is reflected by the amount of AKT phosphorylation.[15] As expected, AKT phosphorylation was significantly induced upon CD38 stimulation using the agonistic anti-CD38 mAB IB4 in primary CLL cells, however, adding the SYK inhibitor R406 largely abrogated this effect (Fig 2A, n = 8, p<0.05). Furthermore, we analyzed the expression of the antiapoptotic protein MCL-1. After 24h CD31 ligation, MCL-1 expression was significantly enhanced. Concomitant treatment with R406 completely abolished this effect (Fig 2B, n = 12, p<0.05 and p<0.01). Representative immunoblots are shown in S2A and S2B Fig. To exclude that the observed induction of AKT phosphorylation is due to changes in AKT expression, we included immunoblots for total AKT levels (S2A Fig).

### Enhanced migration in response to CD38 mediated signals is SYK dependent

CD38 stimulation has previously been linked to increased sensitivity towards migratory stimuli.[7,22] To test whether SYK is involved in this process, we analyzed the potential of primary CLL cells to migrate towards a CXCL12 gradient with and without previous stimulation with IB4 in the presence and absence of R406.

**Fig 3:**
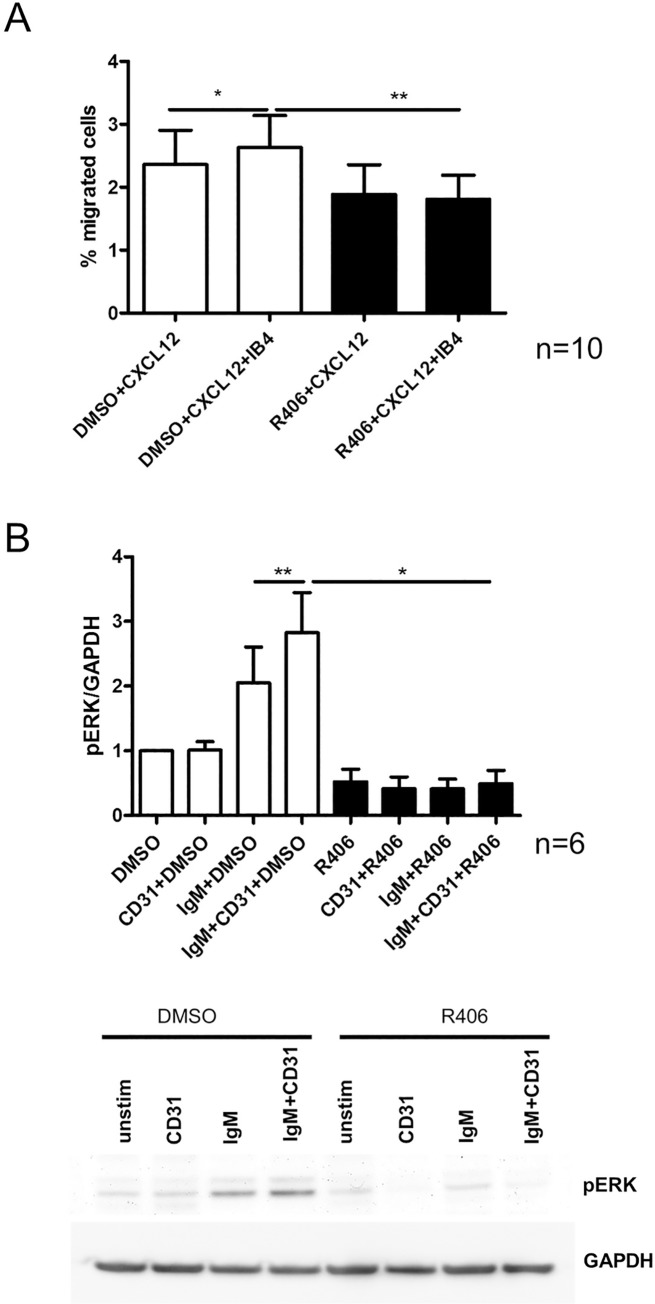
CD38 ligation enhances the migratory potential of CLL cells and is SYK dependent. A) CLL cells were treated with 4 μM R406 or DMSO for 30 min and subsequently stimulated with IB4 for 30 min. Afterwards CLL cells were exposed to a CXCL12 gradient in a transwell chamber (n = 10, p <0.05 and p<0.01). B) Densitometric analysis of western blots for ERK phosphorylation in response to anti IgM stimulation: Before exposure to 5 μg/ml anti IgM for 1 min, CLL cells were treated with 4 μM R406 or vehicle control and prestimulated with CD31 for 15 min (n = 6, p<0.01 and p<0.05). A representative example is shown below.

The results obtained highlighted the presence of a picture where CD38-mediated signals enhance migration of CLL cells and on the contrary, R406 treatment significantly reduced CLL cell migration towards CXCL12 (Fig 3A, n = 10, p<0.05 and p<0.01). A correlation analysis of signalling response and CD38 surface expression showed a slight tendency to a higher migration rate in response to IB4 stimulation in patient samples with higher CD38 expression (relative migration 1.41 (CD38 expression above median) versus 1.25 (CD38 expression below median).

### CD38 enhances BCR-dependent signaling

It has been shown that stimulation of CD38 alone leads to calcium response in several cell types including B cells.[10,14] Moreover, a synergistic effect of CD38 stimulation and BCR expression was reported, leading to an augmentation of BCR signaling capacity.[23] It is widely accepted that BCR signaling plays a crucial role in CLL pathogenesis [24] and that CD38 expression can be associated with a poor outcome of the disease [1], leading to the suggestion that similar mechanisms are active in CLL. To test this hypothesis we analyzed ERK phosphorylation in response to BCR stimulation with or without previous CD31 stimulation. While CD31 short-term stimulation of CLL cells with CD31 for 15 min failed to induce ERK phosphorylation, BCR-dependent ERK phosphorylation was significantly enhanced by concomitant CD38 stimulation (Fig 3B, n = 6, p<0.01). We additionally performed flow cytometry analysis for Ca^2+^ response of primary CLL B cells after induction of CD38 by IB4 stimulation. IB4 alone was not able to induce a detectable response in 6 CLL samples. In contrast, short-term stimulation of CD38 using IB4 for 5 min led to elevated Ca-responses after anti-BCR stimulation in 4 out of 6 patients (S3 Fig).

### SYK inhibition decreases CD38 surface expression on CLL cells

**Fig 4:**
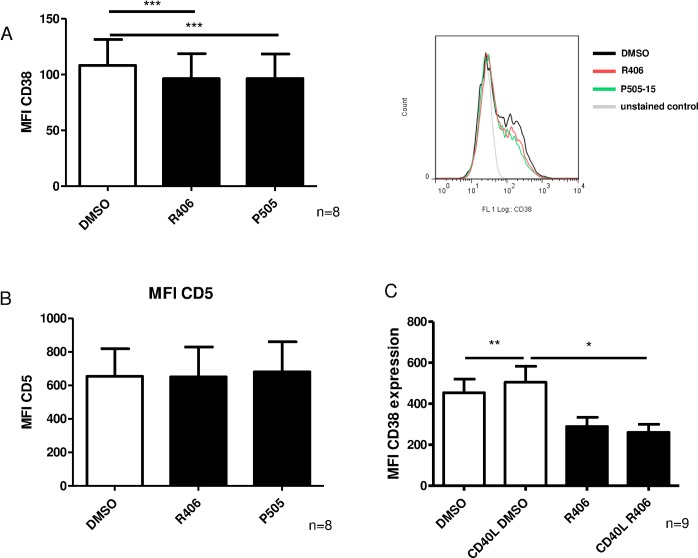
CD38 surface expression is dependent on SYK. Surface expression on CLL cells was analyzed by flow cytometry. A) Analysis of CD38 expression on CLL cells after 24 hs SYK inhibitor treatment (4μM R406 and 2 μM P505-15, respectively) (n = 8, p<0.001). B) Analysis of CD5 surface expression on SYK inhibitor treated CLL cells (n = 8, p = 0.31 and p = 0.42). C) Analysis of CD38 expression after stimulation with CD40L for 24 hs (n = 9, p<0.01 and p<0.05). Error bars indicate SEM.

Next, we tested the influence of SYK inhibitors on CD38 expression and observed significant downregulation of surface CD38 after 24h of treatment with R406 and P505-15, a novel and highly specific SYK inhibitor [25] (Fig 4A, n = 8, p<0.001). The effect was apparently more marked on the subset of CLL cells expressing high levels of CD38. To ensure that this finding reflects a specific down-regulation of CD38 and is not secondary to a general attenuation of expression of surface molecules or to a reduction of the cell size, we analyzed the expression of surface CD5. CD5 expression remained unchanged either after R406 or P505-15 treatments (Fig 4B, n = 8, p = 0.31 and p = 0.42).

CD40 ligation is a known mechanism triggering CD38 expression in proliferative centers, and SYK involvement has been implicated in CD40 signaling.[26,27] Thus we evaluated whether R406 is capable of inhibiting CD38 upregulation induced by CD40L, and detected enhanced CD38 expression after CD40 stimulation that was reversed after co-incubation with R406 (Fig 4C, n = 9, p<0.01 and p<0.05).

**Fig 5:**
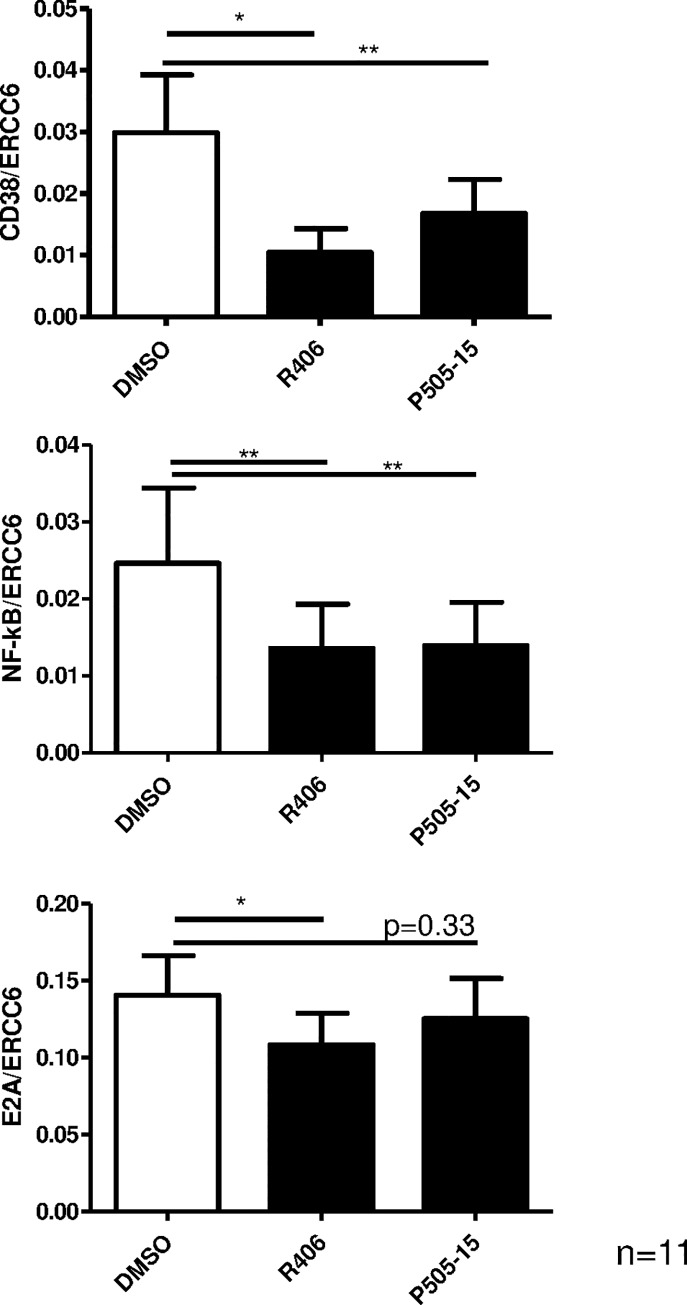
SYK inhibition reduces CD38 surface expression by transcriptional regulation. Primary CLL cells were treated with 4 μM R406 or 2 μM P505-15 for 24 hs. Expression of CD38, NF-kB and E2A mRNA was analyzed by Q-RT-PCR using ERCC6 as a reference. Error bars indicate SEM.

As potential regulators of CD38 expression downstream of SYK, we analyzed the transcriptional levels of NF-kB, CD38 itself and E2A, a bHLH transcription factor involved in the regulation of CD38. Since active NF-kB promotes its own expression, we used NFKB1 mRNA levels as an indicator of NF-kB activity. SYK inhibition by R406 and P505-15 treatment for 24 h significantly downregulated CD38 mRNA expression (n = 11, p<0.05 and p<0.01), NF-kB (n = 11, p<0.01) and, albeit to a lesser extent, E2A (n = 11, p<0.05 and p = 0.33) (Fig 5). To investigate that our results are indeed SYK-dependent and do not have to be regarded as secondary effects due to apoptosis induction, we assessed the viability of primary CLL cells after incubation with the SYK inhibitor R406 for 2,5 hs and 24 hs. We did not observe any induction of apoptosis after 2,5 hours (viability: 63.1% vs. 63.4%, p = 0.885) and we only observed a very low tendency of apoptosis after 24 hours (viability: 54% vs. 49.2%, p = 0.199) (S4 Fig). Apoptosis induction is usually seen after 48 h treatment.

### CD38 expression predicts efficacy of the SYK inhibitor R406

**Fig 6:**
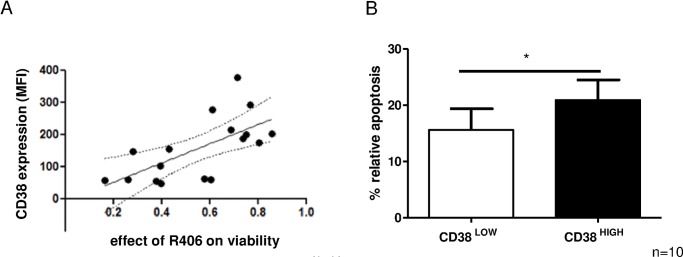
The effect of SYK inhibition on cell viability positively correlates with CD38 expression. A) Primary CLL cells were tested for CD38 surface expression by flow cytometry. Cells were subsequently treated with 4 μM R406 for 24 hs. Induction of apoptosis was analyzed by Annexin V/PI staining. Correlating the rate of apoptosis with CD38 surface expression was analyzed by Spearman rank test (n = 17, p<0.01, Spearman: r = 0.73). B) Primary CLL cells were labeled with APC-labelled anti-CD38 antibody and sorted into CLL cells with CD38^high^ and CD38^low^ expression. Cells were subsequently treated with 4 μM R406 for 24 hs. Apoptosis induction was analyzed by flow cytometry using Annexin V/PI staining (n = 10, p<0.05). Error bars indicate SEM.

To further evaluate the link between CD38 expression and SYK inhibition, we correlated the CD38 expression levels determined by flow cytometry with the effect of R406 on cell viability. Interestingly, R406 was most effective in CLL samples characterized by high expression of CD38 (Fig 6A, n = 17, p<0.01).

High CD38 expression represents an unfavorable state of disease, being associated with high sensitivity towards SYK inhibition rather than directly functionally associated with CLL survival. To this aim, we additionally analyzed whether SYK inhibitor efficacy is directly linked to CD38 expression levels of CLL cells. For this purpose, we sorted CD38^high^ and CD38^low^ CLL cells within individual patients and treated them separately with R406 (4 μM for 24 hs). Our results indicate that CD38^high^ cells exhibit a higher sensitivity towards SYK inhibition as compared to their CD38^low^ counterparts. This is further evidence supporting the view that CD38 mediated signals are involved in SYK-dependent cell survival (Fig 6B, n = 10, p<0.05).

## Discussion

The relevance of CD38 in CLL (other than its being a prognostic marker) has been demonstrated by several studies highlighting its role as both an independent signal transducer and associated factor in a variety of signaling pathways including CXCR4 and BCR signaling. There is a noticeable overlap between CD38- and SYK-mediated pathways, and a functional link between these two proteins has been shown as SYK is phosphorylated upon CD38 ligation in lymphoid cells.[11,14] However the potential link between SYK and CD38 has not yet been addressed in CLL. We therefore investigated the relevance of SYK in CD38-mediated signaling in CLL.

First, we could show that CD38 ligation alone suffices to consistently activate SYK *in vitro*. AKT, a downstream target of SYK, was activated by CD38 ligation, and this induction was disrupted by pharmacological SYK inhibition. Previous studies already identified AKT and ERK as CD38 targets [28,29] and further reports suggest the involvement of Lyn and BTK in CD38 signaling.[30–32] However, our understanding of the functional consequences of activating these signaling molecules downstream of CD38 is incomplete.[11,33] Our study reveals that SYK acts as a central downstream effector of CD38 signaling and regulates survival-, proliferation-, and migration pathways in CLL.

Part of the effects observed after CD38 ligation in CLL cells and B-cell progenitors that others have reported appear to be linked to CD38's association with components in the BCR complex, and the involvement of SYK supports this view. Our data indicate that CD38 plays an active role in BCR signaling, acting as an auxiliary factor enhancing the activation of downstream signaling pathways after BCR ligation, as indicated by increased Ca^2+^ flux and ERK phosphorylation.

SYK has been shown to be involved in the cellular response to various microenvironmental stimuli independent of BCR engagement, including responses to CXCL12.[34] In CLL, CD38 not only marks cells with high migratory potential, it has also been identified as a modulator of CXCL12 signaling.[7,35] Our study also suggests a BCR-independent link between CD38 and SYK in their response to activation of the CXCL12-CXCR4 axis (Fig 3A).

It is still not clear how CD38 is capable of inducing SYK activation. Several studies have implicated a physical association between CD38 and BCR, integrins and CXCR4 complexes and thereby ITAM containing (co)-receptors via their accumulation within lipid rafts. CD38 is capable of influencing signaling outcome independently of its catalytic activity, potentially acting directly on the aforementioned complexes.[36] There are therefore two potential modes of action for the CD38-mediated induction of intracellular pathways: either it physically stabilizes other receptor complexes, facilitating their signaling capacity and subsequently leading to SYK activation, or CD38 directly transduces signals. There are a variety of potential interaction partners for CD38 within both the BCR and CXCR4 complex. These include matrix metalloproteinases, integrins, and others.[37,38] Further investigations are required to understand the consequences of the interactions of CD38 with these signaling complexes in order to understand the molecular mechanism of CD38-mediated intracellular signals.

Our investigation revealed an overt SYK-dependent induction of proliferation signals upon CD38 ligation, yet the effect on actual proliferation of primary CLL cells *in vitro* was low, as assessed by WST-1 assay (data not shown). CLL cell proliferation, however, is highly dependent on the presence of a variety of stimulatory signals, and cellular contact and CLL cells tend to reveal negligible proliferative capacity outside their microenvironmental niches [39,40]. SYK activation by CD38 ligation therefore induces proliferation pathways but requires additional microenvironmental stimulation to overcome G0/G1 arrest.

Interestingly, we also observed that SYK activity is involved in regulating CD38 expression. The CD40-mediated induction of CD38 surface expression [27] was completely blocked by SYK inhibition (Fig 4C). In addition, SYK inhibition led to a significant downregulation of CD38 protein and mRNA expression. We found evidence that both E2A and NF-kB are mediators of CD38 transcription,[41,42] downstream of SYK, in primary CLL cells (Fig 5).

Collectively, our data demonstrate that CD38 plays a functional role in CLL that is linked to SYK, which is activated as a central element in signal transduction and provides a direct link between CD38 expression and CLL cells' sensitivity to SYK inhibition. This is especially interesting when considering an SYK-based targeted therapy in CLL, since CD38 expression serves as both a negative prognostic marker and predicts poor efficacy of standard chemotherapy.[43] A SYK-targeted treatment might therefore be a valuable option when treating this subgroup of CLL patients presenting accelerated disease progression, not only by targeting the activated BCR pathway, but also by reducing CD38-mediated microenvironmental stimuli. Very interestingly, a clinical trial of entospletinib, a selective SYK inhibitor, is now being conducted in CLL.[18]

Considering the functional relevance of CD38 in CLL, CD38 inhibition could also be a promising therapeutic approach to treat patients with CD38-positive CLL. Indeed, novel therapeutic antibodies against CD38 are now being investigated in clinical trials for several hematologic malignancies including CLL. Initial phase I/II studies using the monoclonal antibody daratumumab have already revealed encouraging results in the treatment of multiple myeloma.[44] Daratumumab and SAR650984, another anti-CD38 antibody being investigated in clinical trials, induce strong antibody-dependent cellular cytotoxicity and complement-dependent cytotoxicity, but also seem to functionally interfere with CD38 signaling.[45,46]

Taken together, our findings demonstrate that SYK is an important downstream target of CD38 in mediating proliferation, survival, and migration signals and by acting as a strong regulator of CD38 expression. We therefore propose that disrupting the CD38-SYK axis may represent a promising therapeutic option in CLL. Our findings highlight SYK's pleiotropic function in CLL that helps to explain the clinical success of its inhibition.[17]

## Supporting Information

S1 Fig Western Blots of pSYK (Y525/526) after CD31 ligation in the presence or absence of R406 in two different CLL patients. (JPG)

S2 Fig (A) Representative Western Blot analysis of pAKT, total AKT and β-Actin after CD38 stimulation with IB4 in absence or presence of R406. (B) Representative Western Blot analysis of MCL-1 expression or β-Actin after 24 h of CD38 stimulation with IB4 or CD31 ligation with or without concomitant SYK inhibition.
(JPG)

S3 Fig Ca^2+^ flux of CLL cells was analyzed by flow cytometry using FuraRed. R406 or DMSO-treated cells were incubated with IB4 or PBS for 5 min during continuous Ca^2+^ measurement followed by BCR stimulation as indicated by black arrows.
(JPG)

S4 Fig Viability (Y-axis in %) after 2,5 h (left) or 24 h (right) treatment with 4 μM R406 in 4 CLL patients. (JPG)
