# Supplementary figures and images for: Spleen Tyrosine Kinase Is Involved in the CD38 Signal Transduction Pathway in Chronic Lymphocytic Leukemia
Source: PLoS One. 2016 Dec 30;11(12):e0169159. doi: 10.1371/journal.pone.0169159 (PMC5201248; doi:10.1371/journal.pone.0169159)

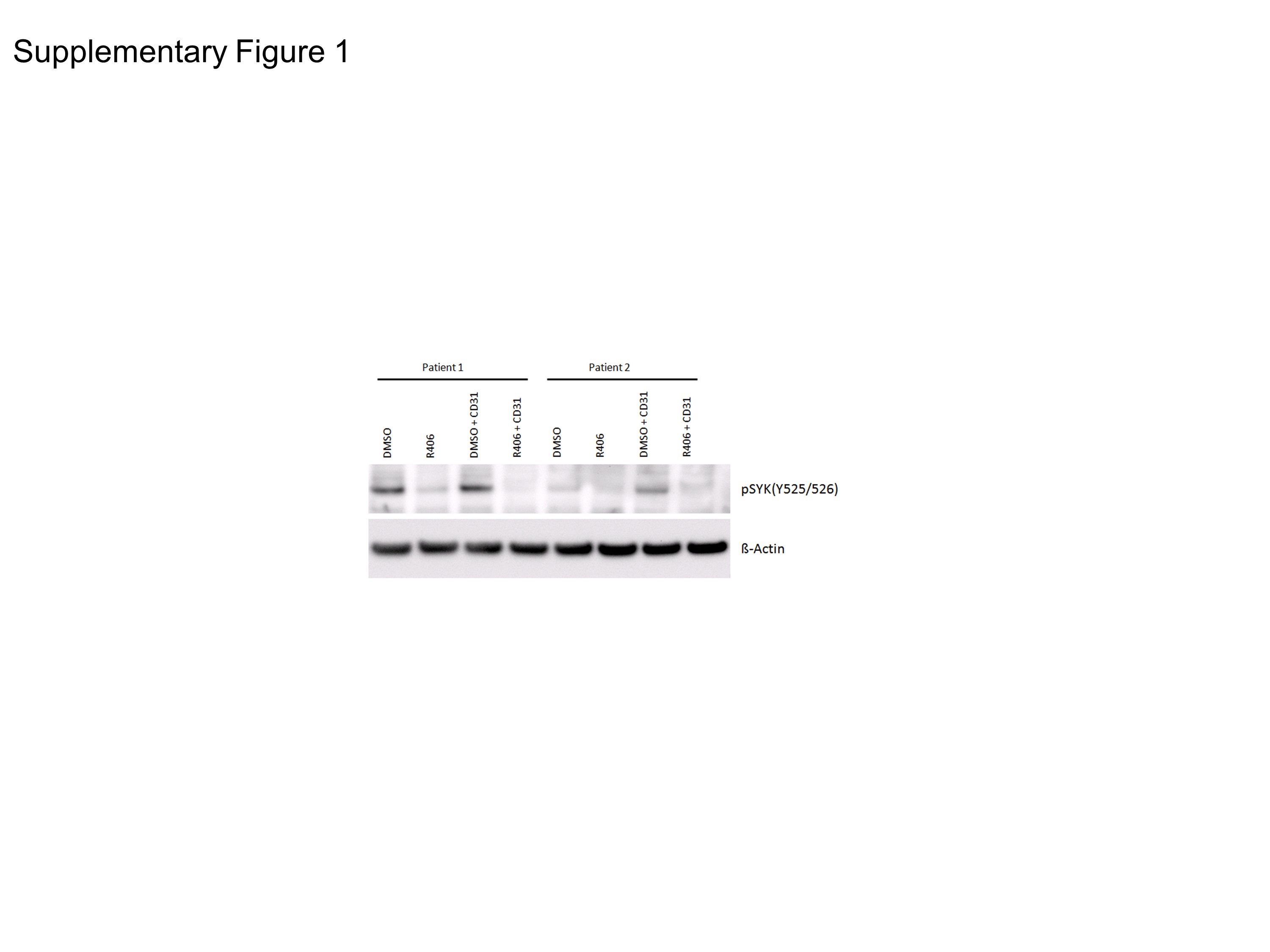

Supplement: S1 Fig — (JPG) [file pone.0169159.s001.JPG]

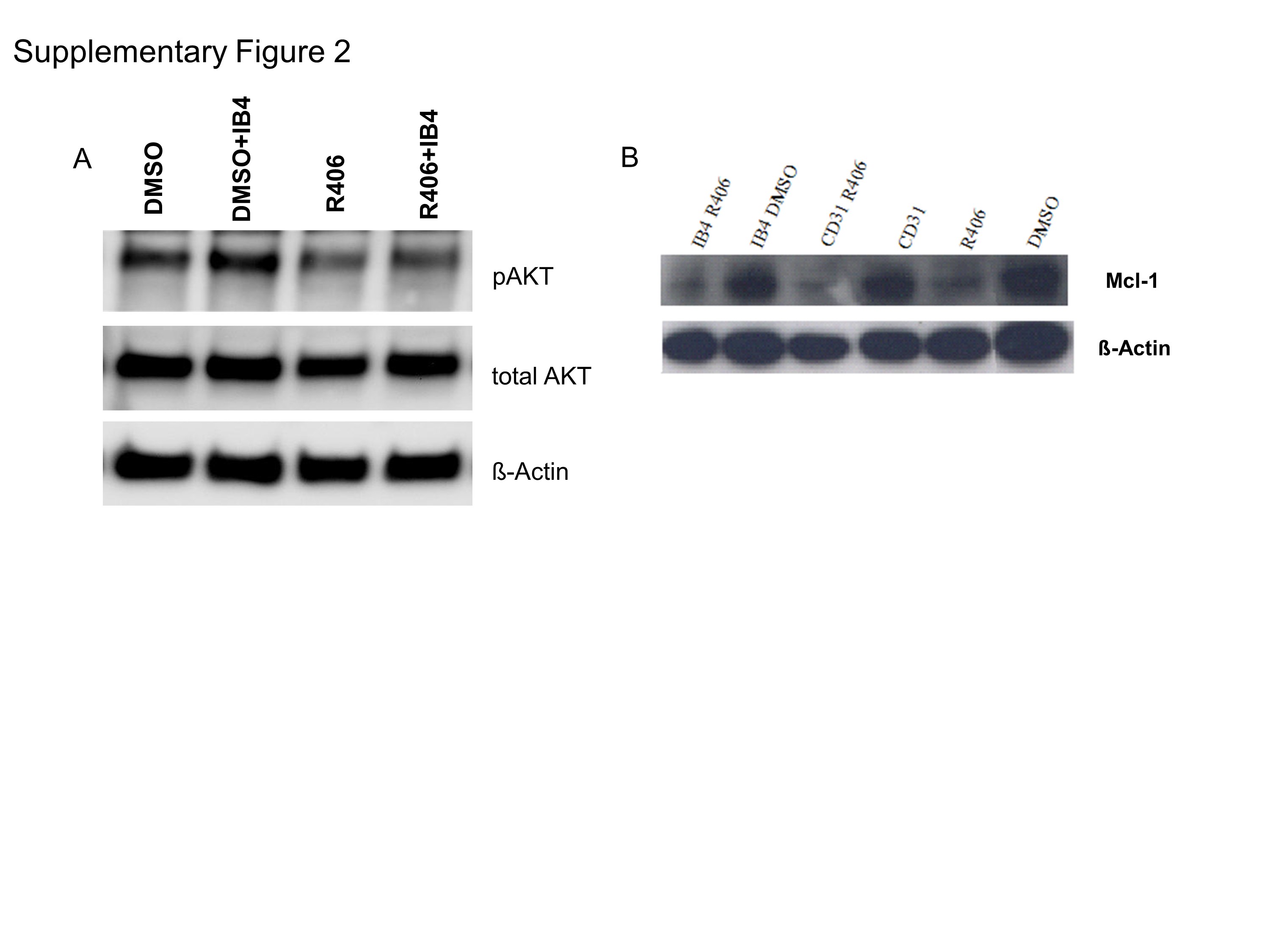

Supplement: S2 Fig — (A) Representative Western Blot analysis of pAKT, total AKT and β-Actin after CD38 stimulation with IB4 in absence or presence of R406. (B) Representative Western Blot analysis of MCL-1 expression or β-Actin after 24 h of CD38 stimulation with IB4 or CD31 ligation with or without concomitant SYK inhibition. (JPG) [file pone.0169159.s002.JPG]

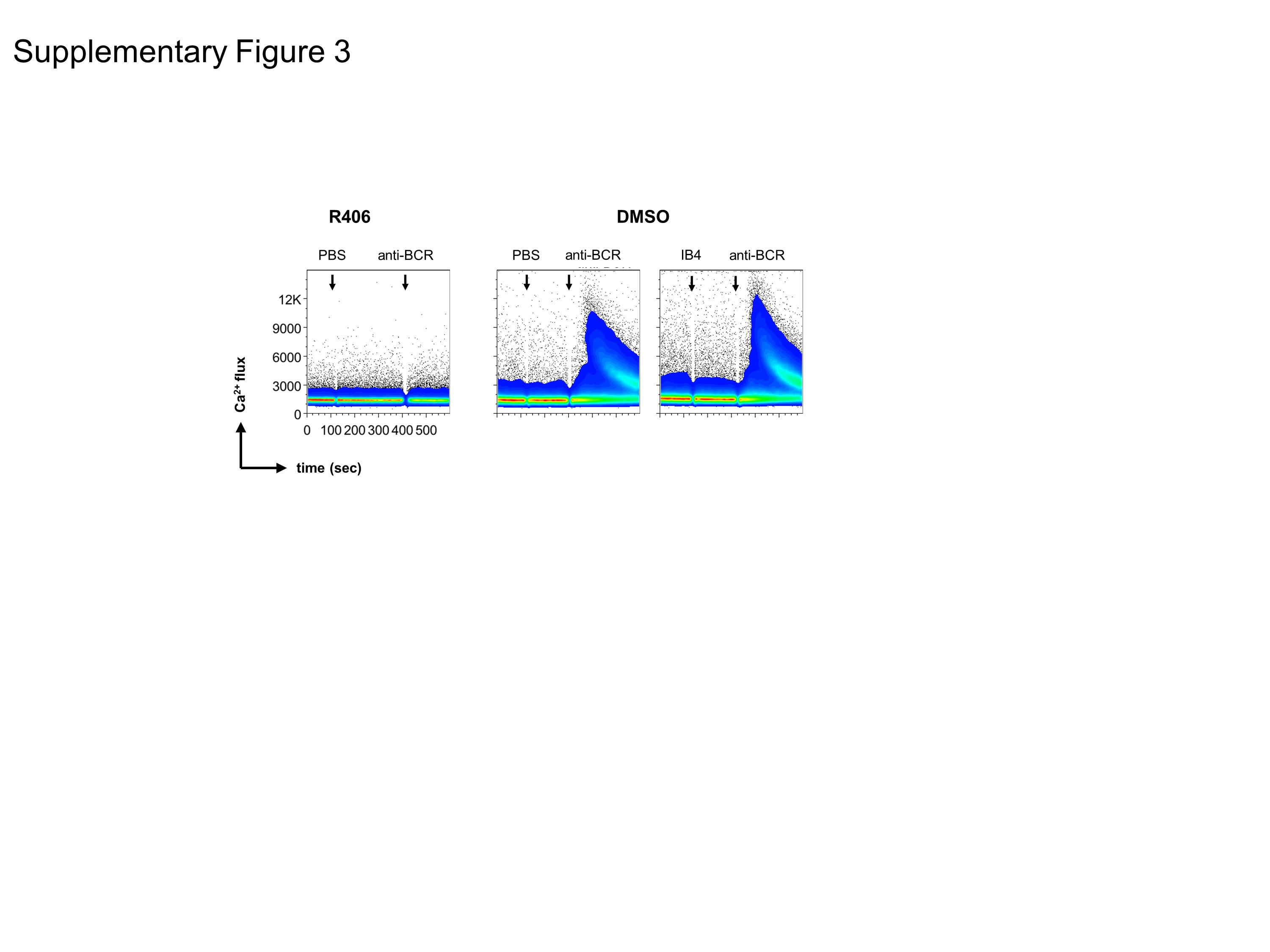

Supplement: S3 Fig — R406 or DMSO-treated cells were incubated with IB4 or PBS for 5 min during continuous Ca2+ measurement followed by BCR stimulation as indicated by black arrows. (JPG) [file pone.0169159.s003.JPG]

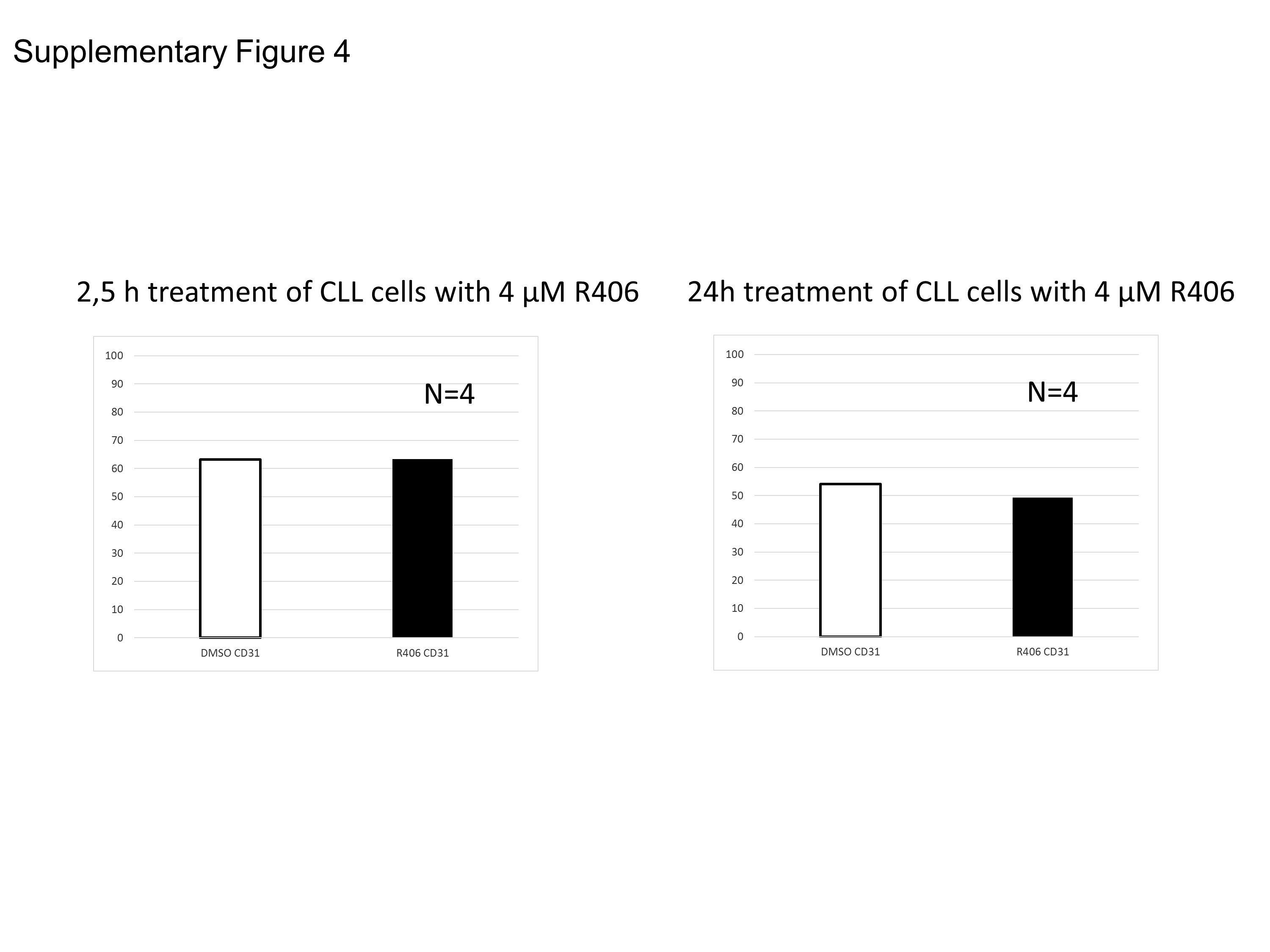

Supplement: S4 Fig — (JPG) [file pone.0169159.s004.JPG]
